# Identification of a Novel Cathelicidin from the *Deinagkistrodon acutus* Genome with Antibacterial Activity by Multiple Mechanisms

**DOI:** 10.3390/toxins12120771

**Published:** 2020-12-04

**Authors:** Lipeng Zhong, Jiye Liu, Shiyu Teng, Zhixiong Xie

**Affiliations:** Hubei Key Laboratory of Cell Homeostasis, College of Life Sciences, Wuhan University, Wuhan 430072, China; zhongliborn@whu.edu.cn (L.Z.); jiyeliu-doc@whu.edu.cn (J.L.); tengshiyu@whu.edu.cn (S.T.)

**Keywords:** *D. acutus* cathelicidin, bacterial cell integrity, antimicrobial peptide, DNA binding

## Abstract

The abuse of antibiotics and the consequent increase of drug-resistant bacteria constitute a serious threat to human health, and new antibiotics are urgently needed. Research shows that antimicrobial peptides produced by natural organisms are potential substitutes for antibiotics. Based on *Deinagkistrodon* *acutus* (known as five-pacer viper) genome bioinformatics analysis, we discovered a new cathelicidin antibacterial peptide which was called FP-CATH. Circular dichromatic analysis showed a typical helical structure. FP-CATH showed broad-spectrum antibacterial activity. It has antibacterial activity to Gram-negative bacteria and Gram-positive bacteria including methicillin-resistant *Staphylococcus aureus* (MRSA). The results of transmission electron microscopy (TEM) and scanning electron microscopy (SEM) showed that FP-CATH could cause the change of bacterial cell integrity, having a destructive effect on Gram-negative bacteria and inducing Gram-positive bacterial surface formation of vesicular structure. FP-CATH could bind to LPS and showed strong binding ability to bacterial DNA. In vivo, FP-CATH can improve the survival rate of nematodes in bacterial invasion experiments, and has a certain protective effect on nematodes. To sum up, FP-CATH is likely to play a role in multiple mechanisms of antibacterial action by impacting bacterial cell integrity and binding to bacterial biomolecules. It is hoped that the study of FP-CATH antibacterial mechanisms will prove useful for development of novel antibiotics.

## 1. Introduction

The use of antibiotics is an effective treatment for infectious diseases, but in recent years, the misuse of antibiotics has caused more bacteria to become resistant or become superbugs [1]. Antimicrobial resistance (AMR) is a broad term referring to resistance to drugs that treat infections caused by bacteria. According to research reports, at the current growth rate, AMR could cause 10 million deaths by 2050, and exceed the 8.2 million cancer deaths in 2019 [2]. Perhaps in the near future, there may be no effective antibiotics available to humans, and more people will die from bacterial infections. Antibiotic resistance has become one of the world’s most pressing health problems.

Antimicrobial peptides (Amps) are small molecular peptides with biological activity that exist in many organisms. The molecular weight of antimicrobial peptides is about 2000~7000 Da, and they are composed of 20~60 amino acid residues [3]. These active peptides are generally characterized by strong alkalinity, thermal stability, and broad-spectrum antibacterial activity [4,5,6,7]. Antimicrobial peptides are an important defense against the invasion of pathogenic microorganisms.

Cathelicidins are one of the largest families of structurally diverse antimicrobial peptides and are at least 400 million years old [8,9]. They were first identified in mammalian myeloid cells, and were then found in a variety of animals, including mammals, fish, birds, amphibians, and reptiles [10]. Snake cathelicidins were found in both venomous and non-venomous snakes in the literature, including KR(F/A)KKFFKK(L/P)K conservative motif [11,12]. They showed a certain efficacy in killing pathogenic microorganisms, but the specific mechanism is not very clear.

*D. acutus* is endemic to southern China; it is also known as the five-pacer viper, commonly used in traditional Chinese medicine. However, to date no cathelicidin has been identified from *D. acutus*. This article is based on *D. acutus* genome bioinformatics analysis, which revealed a new member of the snake cathelicidin family called FP-CATH. We showed that it possesses potent broad-spectrum and rapid antimicrobial activity, low hemolytic toxicity, and strong antibacterial effect on bacterial cell integrity. We also discovered it could bind to bacterial LPS and DNA at low concentrations. In vivo, FP-CATH can protect nematodes in bacterial invasion experiments. Insights into the mechanism employed by FP-CATH will help to fully understand the antibacterial mechanism and guide the development of efficient antibiotics.

## 2. Results

### 2.1. Prediction and Analysis of FP-CATH in the D. acutus Transcriptome and Genome

**Figure 1:**
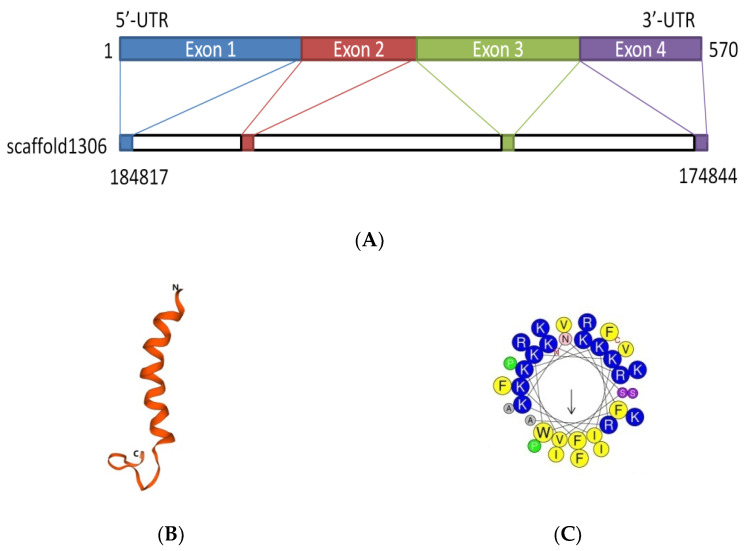

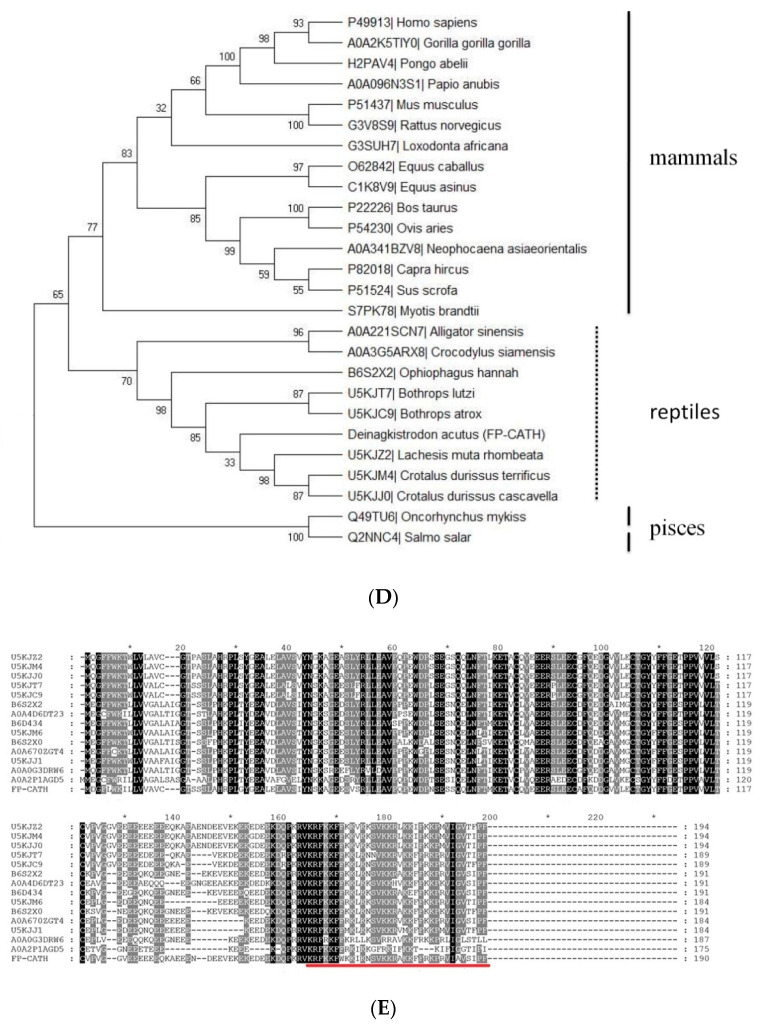
The FP-CATH bioinformatics analysis. (**A**) FP-CATH chromosome localization. (**B**) Tertiary structure prediction of FP-CATH. (**C**) Properties of selected amino acid residues forming the spiral structure of FP-CATH were calculated by Heli-Quest Web server. Arrow means (μH) vector (hydrophobic moment of peptide). (**D**) Phylogenetic analysis of FP-CATH. The tree was constructed using the neighbor-joining method by 1000 bootstrap replicates based on the immature cathelicidins. (**E**) Amino acid sequences align between FP-CATH and other snake cathelicidins.

The *D. acutus* transcriptome and genome were analyzed by local BLAST program in NCBI. The sequence with the highest similarity peptide blast ExPASy was selected (see www.expasy.org). The results showed that it had a similarity to cathelicidin and had never been reported before; it was named FP-CATH. FP-CATH premature peptide was used as a template for genomic localization. It is composed of four exons and three introns and locates at genomic scaffold 1306 (Figure 1A).

Predicting and modeling of FP-CATH secondary structure was conducted using ExPASy. The predicted secondary and tertiary structures of the protein showed that the amino acids at 1-20 of N fragment were typical helical structures as in Figure 1B. The properties of selected amino acid residues forming the spiral structure of FP-CATH were calculated by Heli-Quest Web server. Yellow and gray represent the hydrophobic amino acids, and blue and purple represent the hydrophilic amino acids. Cationic amino acids (lysine and arginine residues, indicated in blue) are abundant on one side of the AMPs (Figure 1C). FP-CATH shows a relatively high isoelectric point of 12.5 in the snake cathelicidin family as per Table 1. The evolutionary tree showed that FP-CATH is closest to vipers among reptiles as per Figure 1D. The premature FP-CATH maximum similarity with *Lachesis muta rhombeata* cathelicidin (ExPASy ID: U5KJZ2) was 82.5%, but mature FP-CATH similarity was 70.5%. Interestingly, the similarity between mature FP-CATH and OH-CATH (ExPASy ID: B6S2X2) was up to 88.2% despite being in different families. It is mainly composed of highly conserved cathelicidin regions with N short signal peptides and C-terminal mature peptides. FP-CATH contains 34 amino acids, and the amino acid at position 7 of the N segment is tryptophan (Figure 1E).

### 2.2. Determination of FP-CATH by Circular Dichromatic Chromatography

**Figure 2:**
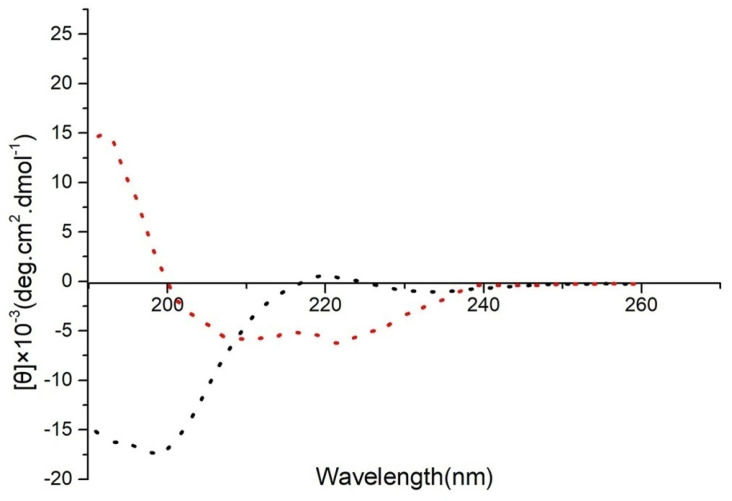
Secondary structure of FP-CATH was analyzed by CD spectroscopy. Red dotted line refers to FP-CATH in 25 mM SDS (membrane mimic condition), black dotted line refers to FP-CATH in PBS buffer.

To confirm the accuracy of the predicted structures, CD spectroscopy was used to detect the secondary structures of 20 μM FP-CATH in PBS buffer. The α-helix structure has a positive spectral band near 192 nm and two negative characteristic acromion spectral bands at 222~208 nm in 25 mM SDS (membrane mimic condition) as per Figure 2.

### 2.3. Determination of MIC of FP-CATH

Through the minimum inhibitory concentrations (MIC) test, FP-CATH showed an antibacterial effect on Gram-negative and Gram-positive bacteria, and the MIC concentration ranged from 1.56 to 12.5 μg/mL as per Table 2.

### 2.4. Hemolytic Activity of FP-CATH on hRBC and Effect of Trypsin on the Antibacterial Activity of FP-CATH

**Figure 3:**
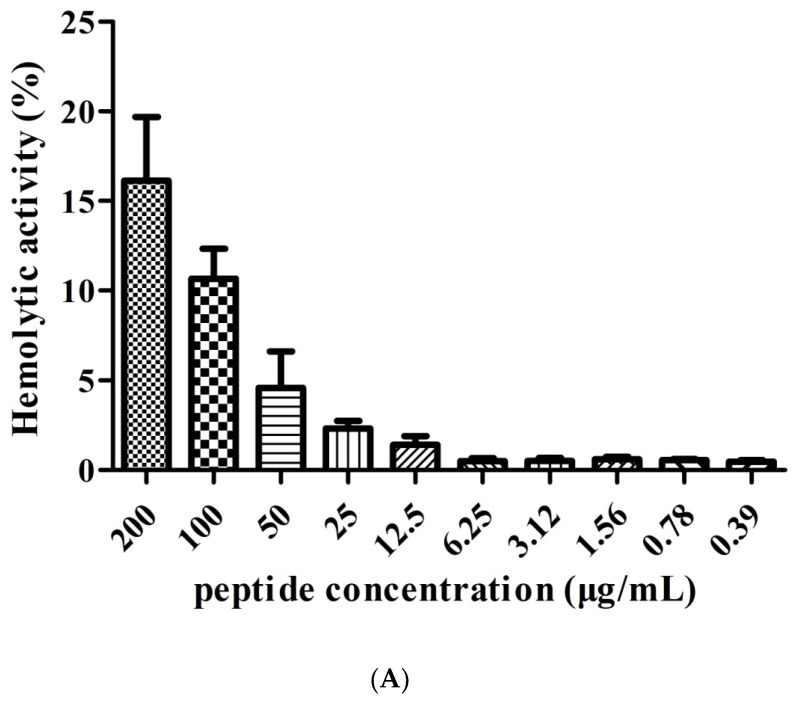

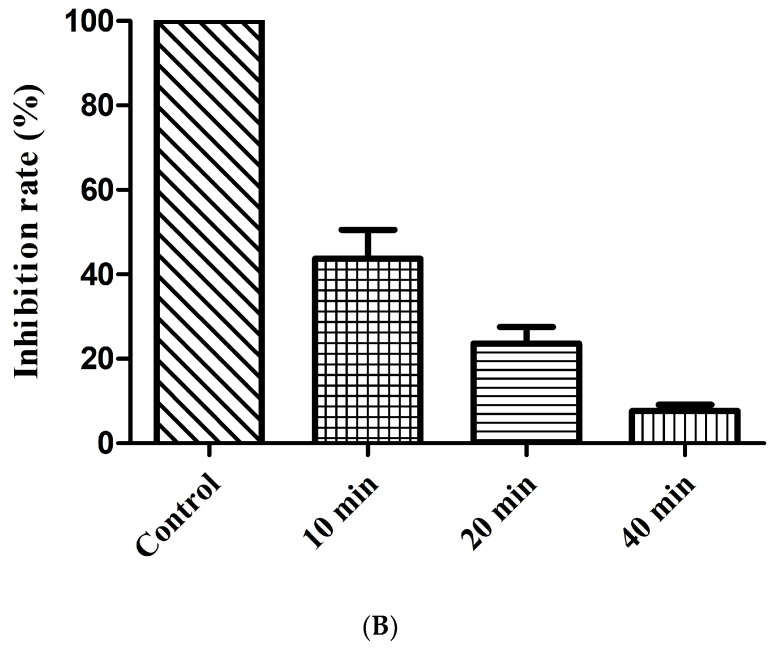
(**A**) hRB Chemolytic activity with FP-CATH. Different concentrations of FP-CATH from 200 to 0.39 μg/mL were detected with hRBC. (**B**) Effect of trypsin on the antibacterial activity of FP-CATH by duration.

The hemolytic activity of FP-CATH on hRBC was measured at different gradient concentrations. The hemolytic activity of FP-CATH was effective at low concentrations (Figure 3A). FP-CATH is sensitive to trypsin as per Figure 3B.

### 2.5. FP-CATH Killing Curve

**Figure 4:**
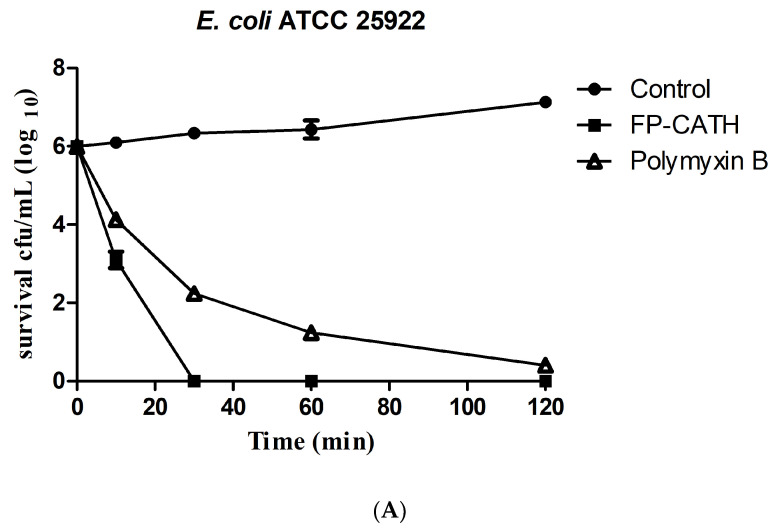

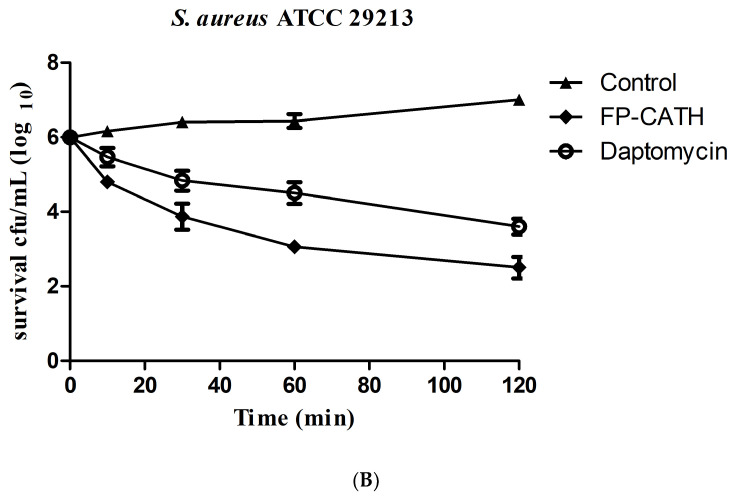
Bacteria time killing curve with FP-CATH. (**A**) *E. coli* ATCC 25922 with 2×MIC FP-CATH, Polymyxin B (2 μg/mL) as positive control. (**B**) *S. aureus* ATCC 29213 with 2×MIC FP-CATH. Daptomycin (4 μg/mL) as positive control. At different times: 0, 10, 30, 60 and 120 min.

Exposed to 2×MIC, FP-CATH showed rapid bactericidal activity on the growth of *E. coli* ATCC 25922. The living cells of *E. coli* ATCC 25922 decreased significantly in 10 min as per Figure 4A. *S. aureus* ATCC 29213, showed slower bactericidal activity. It needed 120 min for less than log 2 CFU/mL (Figure 4B).

### 2.6. FP-CATH Binding of LPS

**Figure 5:**
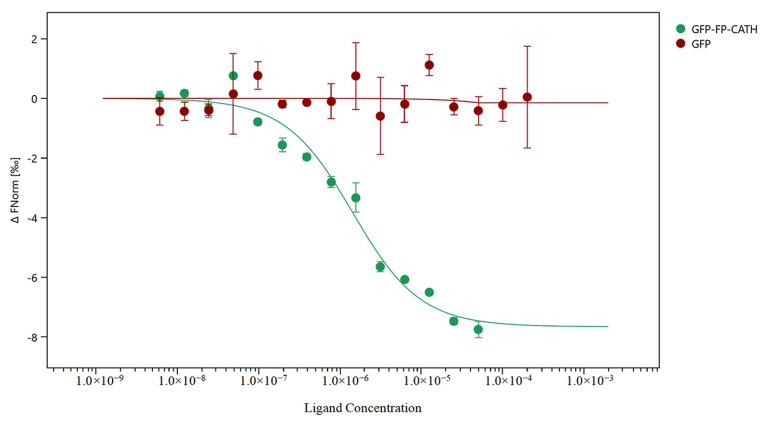
Using of microscale thermophoresis (MST) test the binding ability of GFP-FP-CATH with LPS. The *X* axis represents LPS concentrations, and the *Y* axis represents the fraction of the change in fluorescence.

The microscale thermophoresis (MST) technique was used to assess whether direct binding of LPS to FP-CATH occurred by using a series of LPS to 1 μM GFP-FP-CATH. When LPS was binding of GFP-FP-CATH, the thermal swimming signal was changed, and GFP as control showed almost no signal change with LPS as shown in Figure 5. The K_D_ of GFP-FP-CATH was 1.31 ± 0.30 μM compared to GFP, which provided partial evidence for binding of LPS to FP-CATH.

### 2.7. Effect of FP-CATH on Morphology of Bacterial Cells

**Figure 6:**
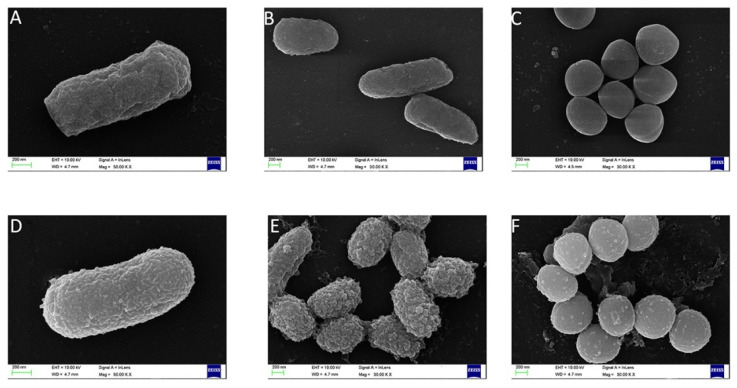
Scanning electron microscopy was used to observe the change of bacteria morphology. Without peptide: (**A**) *E. coli* BL21, (**B**) *P. donghuensis* HYS, (**C**) MRSA 315838; with FP-CATH: (**D**) *E. coli* BL21, (**E**) *P. donghuensis* HYS, (**F**) MRSA 315838.

Scanning electron microscopy results showed that FP-CATH induced certain surface changes in *E. coli* BL21, *P. donghuensis* HYS, and MRSA 315838 at 5×MIC for 60 min. The morphology of bacterial cells exposed to FP-CATH showed significant changes that were manifested as cell deformation and formation of vesicles (Figure 6).

**Figure 7:**
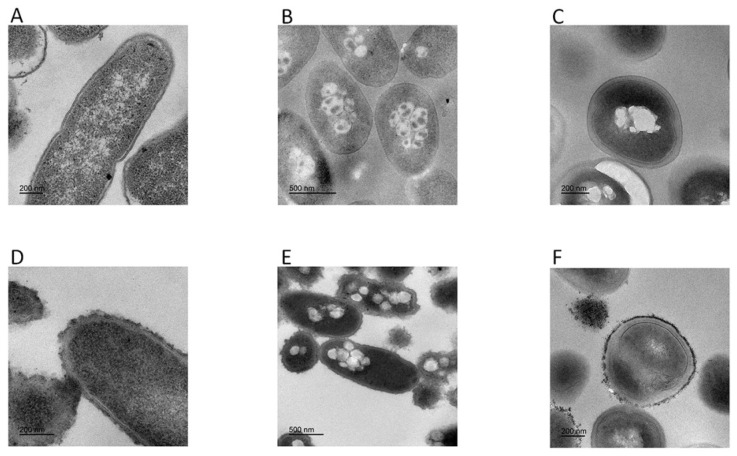
Transmission electron microscopy was used to observe change of bacteria morphology. Without peptide: (**A**) *E. coli* BL21, (**B**) *P. donghuensis* HYS, (**C**) MRSA 315838; with FP-CATH: (**D**) *E. coli* BL21, (**E**) *P. donghuensis* HYS, (**F**) MRSA 315838.

Transmission electron microscopy results showed morphological and intracellular changes of bacteria treated with FP-CATH at 5×MIC for 60 min. Peptide-treated *E. coli* BL21, *P. donghuensis* HYS, and MRSA 315838 cells showed significant irregular arrangement of surface and cytoplasmic inclusion release. The cytoplasmic content of the peptide-treated cells showed more aggregation and condensation than control cells (Figure 7).

### 2.8. P. donghuensis HYS Cell Integrity by FP-CATH

**Figure 8:**
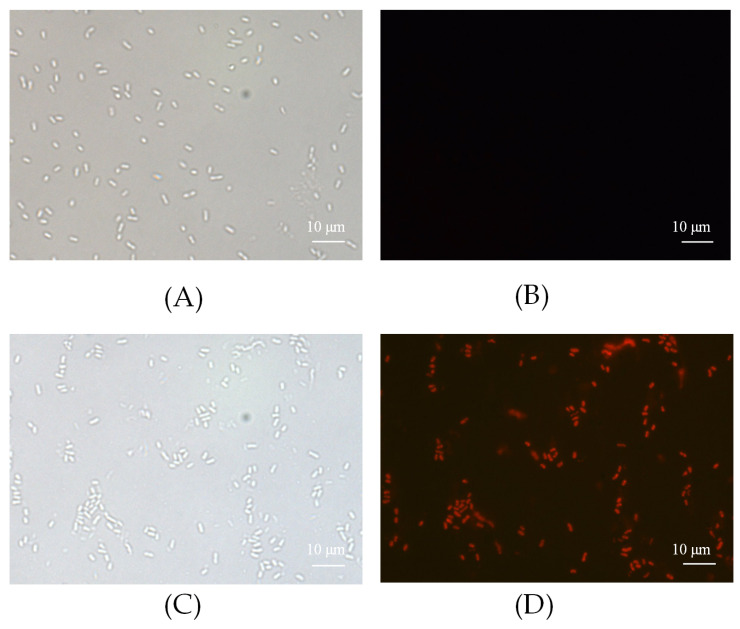
Bacteria cell integrity was detected by fluorescence PI. (**A**,**B**) Control without peptide; (**C**,**D**) *P. donghuensis* HYS with FP-CATH.

PI was used to detect the integrity of the bacterial membrane. FP-CATH damaged the integrity of *P. donghuensis* HYS, as the fluorescence intensity of PI increased after incubation for 10 min. PI with positive staining was almost 80%, indicating that bacterial membrane integrity was impaired (Figure 8).

### 2.9. P. donghuensis HYS Genomic Block by FP-CATH

**Figure 9:**
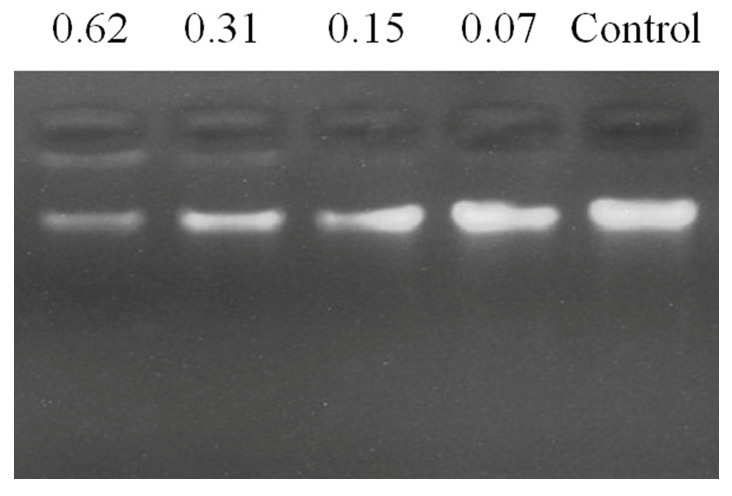
Gel block analysis of peptide binding to *P. donghuensis* HYS genome DNA, indicating the weight ratio above each lane (peptide: DNA).

To assess the DNA binding activity of FP-CATH, electrophoretic mobility was determined. The results showed that FP-CATH could interact with *P. donghuensis* HYS genomic DNA. The DNA incubated with different concentrations of peptides migrated different distances than the DNA itself. For FP-CATH, the electrophoretic mobility of DNA can be inhibited by a peptide: DNA weight ratio of 0.31:1 as per Figure 9.

### 2.10. C. elegans Infection Protection

**Figure 10:**
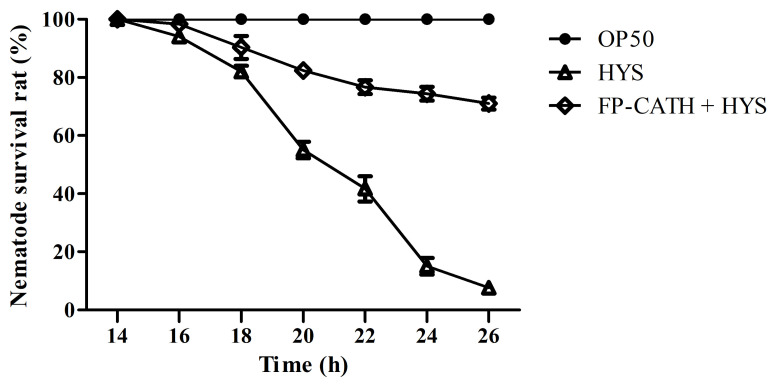
Survival of nematodes in a liquid lethal model infected with *P. donghuensis* HYS. The 96-well plates were incubated at 22 °C. The number of nematode deaths was observed and counted every two hours.

The nematodes immersed in liquid culture of *E. coli* OP50 survived well, and no individual died within 26 h of observation. However, the death rate of nematodes immersed in *P. donghuensis* HYS liquid culture was less than 20% in the first 18 h, and increased rapidly to 90% at 18~22 h. All nematodes died by 26 h, and the dead nematodes remained rigid in the culture hole. However, the 26 h survival rate of the nematodes with 5×MIC FP-CATH was 71 ± 3.6% (Figure 10).

## 3. Discussion

AMPs have been seen for some time as a promising alternative that can curb the rapid spread of antibiotic resistance [14,15]. With the development of technology, bioinformatics has become an important means to discover new antimicrobial peptides [16]. They can be predicted from genomes by bioinformatics analysis, but their exact structure and function require validation. In this study, a new antimicrobial peptide, named FP-CATH, was identified by genome analysis of *D. acutus*. ExPASy protein tools prediction and circular dichroism were used to study its physical and chemical properties, and showed a typical α helical structure. It showed a tryptophan mutation in the N-terminal conserved region compared with traditional snake cathelicidin by alignment. Previous studies have shown that the aromatic side chains of tryptophan can affect the interface of lipid bilayer, disturb the internal structure of cell membrane, and promote the anchoring of AMPs on bacterial membrane [17]. This may partly explain why FP-CATH has a broad spectrum of antibacterial activity.

Polymyxin B is known to be sensitive to Gram-negative bacteria, and daptomycin is sensitive to Gram-positive bacteria. FP-CATH had no selective antibacterial effect, and affected both Gram-negative bacterial and Gram-positive bacterial integrity, compared with polymyxin B and daptomycin. It has a fast and broad spectrum antibacterial activity. AMPs are attracted to the bacterial surface by electrostatic bonding between the cationic peptide and the surface structure of anionic bacteria, such as LPS in Gram-negative bacteria and lipoteichoicacid (LTA) in Gram-positive bacteria [18]. The membrane insertion and permeability of AMPs are key features of its antibacterial activity. An MST result showed that LPS could bind to GFP-FP-CATH with K_D_ as 1.31 ± 0.30 μM. It was reported that the K_D_ value of Polymyxin B-LPS was 0.5 ± 0.10 μM based on the fluorescence probe detection method [19]. LPS is one of the main components of the outer membrane of Gram-negative bacteria, distinguishing the components of mammalian cells. The higher affinity with LPS indicates that the antimicrobial peptides easily bind to bacteria and accumulate on the membrane. The surface disturbance, the complete change of cellular structure, the production of vesicles, and the release of bacterial contents were observed by SEM and TEM. PI dye could pass through into the bacteria with membranes damaged by FP-CATH.

However, the membrane mechanism of AMPs is not the only way to kill pathogenic microorganisms. Gel blocking experiments showed that antimicrobial peptides could bind to bacterial genomes at a peptide: DNA weight ratio of 0.31:1. Previous studies have shown that short antimicrobial peptides containing WK motifs have an outstanding DNA binding ability [20]. Dating back to the 1970s, there was the first understanding of how peptides containing tryptophan and lysine bind to single or double-stranded DNA [21,22]. Tryptophan and lysine (WK) residues are key components that bind to DNA molecules [23]. Antimicrobial peptides bind to bacterial DNA interfering with replication and affecting normal metabolism there by killing bacteria.

Blood compatibility of antimicrobial peptides is important. FP-CATH has low hemolytic activity to human red blood cells. There are reports in the literature that hemolytic activity of OH-CATH was 10.80% at 200 μg/mL [24]. FP-CATH has a slightly higher hemolytic activity than OH-CATH, which was 16.13 ± 5.1% at 200 μg/mL and similarly with pexiganan which was 16.5% at 200 μg/ml [24]. In general, it is clinically impossible to use such a large dose. The salt concentration has a certain effect on FP-CATH. In the presence of 100 mM NaCl, the MIC value of FP-CATH against *E. coli* ATCC 25922 was 6.25 μg/mL. It was four times higher than without NaCl (data not shown). FP-CATH is also sensitive to trypsin. FP-CATH needs further optimization such as simplification of peptide structure, peptide cyclization, N-terminal and C-terminal modification, and peptide bond modification to enhance stability. The acylation of antimicrobial peptides can improve the antibacterial properties, but also leads to the decrease of blood compatibility [25]. Therefore, it is necessary to find the right balance between antibacterial activity and biocompatibility.

## 4. Conclusions

In this paper, FP-CATH, a new member of cathelicidin family, was discovered from the *D. acutus* genome. Its antibacterial activity was verified and possible antibacterial mechanism was discussed. From the obtained results, FP-CATH induces bacterial death via networks, not by a one single mechanism. It can be speculated that FP-CATH not only affects the complete cellular structure, but also interacts with biological macromolecules such as LPS and nucleic acid, and interferes with the normal metabolism of cells. The new antimicrobial peptides have the potential to act against bacteria through a variety of mechanisms. Overall, our findings in this study will facilitate the development of novel antimicrobial agents with a wide range of clinical and therapeutic applications.

## 5. Materials and Methods

### 5.1. Materials

FP-CATH (KRFKKFWKKIKNSVKKRAKKFFRKPRVIAVSIPF) and FP-CATH DNA sequences (AAACGCTTTAAAAAATTTTGGAAAAAAATTAAAAACAGCGTGAAAAAACGCGCGAAAAAATTTTTTCGCAAACCGCGCGTGATTGCGGTGAGCATTCCGTTT) were synthesized by Wuhan Aoke Ding sheng Biological Company. Propidium iodide (PI), triton X100, polymyxin B, daptomycin, and other organic solvents were all purchased from GOYOO BIOTECH CO (Nanjing, China). All other reagents were of high purity reagent grade.

### 5.2. Bacterial Strains

*E. coli* BL21, *E. coli* ATCC 25922, *P. aeruginosa* ATCC 27853, *S. aureus* ATCC 29213, *P. aeruginosa* ATCC 15692, *P. donghuensis* HYS, and *C. albicans SC* 5314wereobtained from the China Center for Type Culture Collection. *A. baumannii* ATCC 19606 and *A. baumannii* ATCC 17978 were generously provided by Professor Yunsong Yu (Department of Laboratory Medicine, The Second Affiliated Hospital of Zhejiang University School of Medicine, Hangzhou, China). Methicillin-resistant *S. aureus* (MRSA) 315837 and MRSA 315838 were from Wuhan Maternal and Child Health Care Hospital. Wild-type *Caenorhabditis elegans* (Bristol N2) from *Caenorhabditis* Genetics Center were used in this study.

### 5.3. Identification of FP-CATH in D. acutus Genome

NCBI local blast (http://blast.ncbi.nlm.nih.gov) analysis of *D. acutus* transcriptome and genome was used (Bio Project Accession Number: PRJNA314443 and PRJNA314559) [26]. The U5KJT7 (ExPASy ID) cathelicidin as a template gene sequence was compared with the *D. acutus* genome, selecting highly matched sequences (*p* < 0.001). Its transcription protein sequence data were blast with ExPASy uniprot and *D. acutus* transcriptome. Finally, the mature peptide regions of cathelicidin sequences predicted by *D. acutus* were identified and named FP-CATH. The immature protein sequences of FP-CATH were compared with the *D. acutus* genome sequence data set as template sequences for chromosome localization. Predicting and modeling of FP-CATH secondary structure were by ExPASy (www.expasy.org).

### 5.4. Phylogenetic Analysis

Cathelicidin sequences for multiple sequence alignment and phylogenetic analysis were obtained from the ExPASy (www.expasy.org) protein database. MEGA 5.0 was used to construct the phylogenetic tree with immature cathelicidin domains.

### 5.5. Antimicrobial Activity Assay

Minimum inhibitory concentrations (MICs) for each strain were determined in appropriate media using standard continuous dilution by the Clinical and Laboratory Standards Institute (CLSI) standard reference method (document M27-3), with some modifications [27]. Briefly, serial gradient dilutions of FP-CATH were added to the same number of log-phase bacteria in 96-well plates to give a final concentration of 10^5^ CFU/mL. Drug concentrations ranging from 200 to 0.1 μg/mL were incubated with log-phase bacteria at 37 °C or 30 °C for 12 h. *C. albicans* SC 5314 was used in sabouraud liquid medium (1% protein, 4% glucose, 0.01% chloramphenicol) and oscillated at 28 °C. The MIC was defined as the concentration at which the *OD*_600_ of the mixture did not increase against the initial optical density. MBC is the minimum concentration of bacterial cell killing at least 99.9%. Bacteria without any drug treatment were used as control.

### 5.6. Time Killing Curve

The bacterial killing kinetic assay was performed according to a previous method [28]. Briefly, *E. coli* ATCC 25922 and *S. aureus* ATCC 29213 were incubated in Mueller-Hinton broth at 37 °C to exponential phase and diluted to 10^6^ CFU/mL. FP-CATH (2 × MIC, were 3.12 µg/mL for *E. coli* ATCC 25922 and 12.5 µg/mL for *S. aureus* ATCC 29213) added to the bacterial suspension and incubated at 37 °C for 0, 10, 30, 60 and 120 min, respectively. Polymyxin B (2 μg/mL) and Daptomycin (4 μg/mL) were used as positive control. 50 µL of the dilution was extracted at each time point and diluted with fresh broth 1000-fold, and 50 µL of the final dilution was seeded on agar plates. After incubation at 37 °C for 24 h, the viable colonies were determined.

### 5.7. Peptide-Induced Membrane Damage

FP-CATH induced membrane damage assay was measured by the uptake of PI. If the bacterial membrane is damaged, PI can enter through the outer and inner membranes and into the cells. Log-phase *P. donghuensis* HYS cells were resuspended in MHB to 10^6^ CFU/mL with 5 × MIC of FP-CATH, then incubated with 0.1 M PI for 10 min and observed under a fluorescence microscope.

### 5.8. Hemolytic Assay and Effect of Trypsin on the Antibacterial Activity of FP-CATH

Human blood was obtained from a healthy donor at Wuhan University Hospital and approved by the Institutional Review Board. Normal human red blood cells (hRBC) were cleaned with PBS buffer (10mM, pH 7.4) 3 times, and then 5% hRBC suspension was prepared with PBS buffer. The suspension was treated with a series of drugs of different concentrations and pre-cultured in a 37 °C water bath for 15 min. Then the supernatant absorbance was determined at 540 nm after centrifugation at 1000× *g* for 1min.Hemolytic percentage (%) = (*A*_pep_ − *A*_blank_)/(*A*_pos_ − *A*_blank_) × 100. *A*_pos_ with 0.2% Triton X-100 were positive controls and *A*_blank_ with PBS (no FP-CATH) were negative controls [29]. FP-CATH was treated with 1 mg/mL of trypsin solution for different times (10, 20 and 40 min) at 37 °C. After enzyme reaction at 100 °C for 10 min, *E. coli* ATCC 25922 suspension (10^8^ CFU/mL) was added, with FP-CATH final concentration of 5×MIC; this was repeated 3 times. Inhibition rate (%) = (*N*_control_ − *N*_treated_)/*N*_control_ × 100. *N*_control_ means the number of bacteria with PBS, *N*_treated_ is the number of bacteria with FP-CATH treated by trypsin.

### 5.9. Cloning and Expression of GFP-FP-CATH Protein

GFP gene sequence was cloned from lacO/LacI-GFP system [30]. GFP-FP-CATH fusion gene sequence was constructed and inserted into pET-28a as the expression vector. The recombinant plasmid was transformed into *E. coli* DH5α strain receptive cells. The connecting constructed plasmids were transformed into *E. coli* BL21 (DE3) cells. GFP-FP-CATH fusion protein was purified after IPTG-induced expression and detected by electrophoresis.

### 5.10. LPS Binding GFP-FP-CATH Assay

The binding of LPS to FP-CATH was examined by microscale thermophoresis (MST, Monolith NT.115, Nano Temper Technologies, Munich, Germany) as previously described [31]. The GFP-FP-CATH was dissolved in PBS buffer to a constant concentration of 1 μM, and GFP as control under the same conditions. LPS from *E. coli* 0111:B4 (#L2880, Sigma, St. Louis, Missouri, USA) was diluted with PBS buffer in a dilution series with the highest concentration of 200 μM. 10 μL of the different LPS dilutions was mixed with 10 μL of the GFP-FP-CATH solutions. Mixed samples were loaded into glass capillaries and the MST analysis was performed. The MST curves were fitted with a K_D_ method using MO Affinity Analysis software to obtain a K_D_ value for binding between LPS and GFP-FP-CATH.

### 5.11. CD Spectroscopy

CD measurements were performed on a Chirascan Cs/v100 (Applied Photophysics Limited company, Kingston Road, Surrey, UK) using a 1-mm path-length quartz plate at 25 °C. FP-CATH was dissolved in PBS buffer and 25 mM SDS (membrane mimic condition) at a concentration of 20 μM. Each spectrum was obtained by averaging three scans in the 180~260 nm wavelength range; the output pressure of nitrogen was 0.4 Mp, time-per-point was 0.5 s and the results were recorded as the mean residue molar ellipticity (θ).

### 5.12. Scanning Electron Microscopy (SEM)

As previously described [32]. *E. coli* BL21, *P*. *donghuensis* HYS, MRSA 315,838 cells were cultured to mid-log phase and harvested. Bacteria were washed three times with PBS buffer, then resuspended to 0.2 at *OD*_600_. The mixture of bacteria and the peptide (at a concentration of 5×MIC) was incubated at 37 °C for 60 min. Bacteria without peptide were used as control. Bacteria were observed by field emission scanning electron microscopy (QUANTA 200, FEI company, Hillsboro, USA).

### 5.13. Transmission Electron Microscopy (TEM)

As previously described [33]. The structural changes induced by FP-CATH on *E. coli* BL21, *P*. *donghuensis* HYS, and MRSA 315838 were studied using TEM as described. Bacterial cells were suspended in PBS phosphate buffer. The mixture of bacteria and the peptide (at a concentration of 5×MIC) was incubated at 37 °C for 60 min. 3% uranium-lead citrate double staining was used with transmission electron microscope (JEM-1400 plus, Japan Electronics Co. LTD, Shojima, Tokyo, Japan) observation.

### 5.14. DNA Binding Assay

*P*. *donghuensis* HYS genomic materials were extracted with a bacterial genomic DNA extraction kit (Sangon Biotech Co., Ltd., Shanghai, China). Genomic DNA purity was evaluated (OD260/OD280 = 1.89). Genomic DNA concentration was determined by measuring absorbance at 260 nm (NanoDrop 2000, Thermo, San Jose, CA, USA) at room temperature. Genomic DNA (200 ng) was incubated with FP-CATH by peptide/DNA weight ratio (0.62, 0.31, 0.15 and 0.07:1) at room temperature for 10 min and assayed on a 1% agarose gel to detect DNA binding [7].

### 5.15. Caenorhabditis elegans Infection Assay

In vivo, nematode models were used to evaluate antimicrobial potential [34,35,36,37,38]. *P*. *donghuensis* HYS was used with strong virulence towards *C*. *elegans* [39,40]. *E. coli* OP50 and *P*. *donghuensis* HYS strains were inoculated into a 5 mL LB liquid medium and incubated overnight. Centrifugal collection of bacteria with heavy suspension M9 solution and dilution to *OD*_600_ = 0.05, including the cholesterol solution to concentration of 5 μg/mL was conducted. Using three repeat holes, *P*. *donghuensis* HYS and *E. coli* OP50 strains of bacteria were packed into 96-well plates at 100 μL/hole. L4 *C. elegans* Bristol N2 were washed with M9 Buffer from the NGM plate and allowed to settle spontaneously. After the supernatant was removed, the *C. elegans* Bristol N2 were rinsed with M9 Buffer 3 times. Then, the nematodes were transferred to the 96-well plates with about 20 nematodes per well. The 96-well plates were incubated in a 22 °C incubator, and the number of nematode deaths was observed and counted every two hours.

### 5.16. Statistical Analysis

The results (mean ± SD) were statistically analyzed by one-way ANOVA with repeated measures used to analyze factors influencing the size of the growth inhibition zone.

## Figures and Tables

**Table 1:** The physical and chemical properties of snake cathelicidins.

Cathelicidin	*pI*	*Mw*
FP-CATH	12.50	4236.30 (This study)
B6S2X2(OH-CATH)	12.34	4155.23 [13]
U5KJZ2	12.09	4186.38 [11]
B6D434	12.04	4198.25 [5]
A0A4D6DT23	11.65	4150.29 (ExPASy)

pI and Mw were calculated by ExPASy-Compute pI/Mw tool.

**Table 2:** Antimicrobial activity of FP-CATH.

	MIC (μg/mL)			MBC (μg/mL)
Bacteria	FP-CATH	Polymyxin B	Daptomycin	FP-CATH
Gram-negative bacteria				
*E. coli* ATCC 25922	1.56	0.5	–	3.12
*E. coli* BL21	6.25	1	–	12.5
*A. baumannii* ATCC 19606	12.5	2	–	12.5
*A. baumannii* ATCC 17978	6.25	0.5	–	12.5
*P. aeruginosa* ATCC 27853	3.12	0.5	–	3.12
*P. aeruginosa* ATCC15692	6.25	0.5	–	6.25
*P. donghuensis* HYS	6.25	1	–	6.25
Gram-positive bacteria				
*S. aureus* ATCC 29213	6.25	–	0.25	12.5
*S. aureus* MRSA 315837	12.5	–	1	25
*S. aureus* MRSA 315838	6.25	–	1	12.5
Fungi				
*C. albicans* SC-5314	25	ND ^a^	ND	50

ND ^a^, not determined; “–”, not sensitive.
